# The enigma of soluble LDLR: could inflammation be the key?

**DOI:** 10.1186/s12944-020-1199-9

**Published:** 2020-02-03

**Authors:** Majambu Mbikay, Janice Mayne, Michel Chrétien

**Affiliations:** 1 Functional Endoproteolysis Laboratory, Clinical Research Institute of Montreal, 110 avenue des Pins Ouest, Montréal, Québec, H2W 1R7 Canada; 2 Chronic Disease Program, Ottawa Hospital Research Institute, Ottawa, ON Canada; 3 Department of Biochemistry, Microbiology and Immunology, University of Ottawa Faculty of Medicine, Ottawa, ON Canada

**Keywords:** Soluble LDLR, Ectodomain shedding, ADAM-17, Inflammation

## Abstract

Soluble low-density lipoprotein receptor (sLDLR) is the circulating ectodomain of transmembrane LDLR. Its blood level strongly correlates with that of triglycerides (TG). This correlation has eluded satisfactory explanation. Hypertriglyceridemia and shedding of the ectodomain of many transmembrane receptors often accompany inflammatory states. The shedding mostly occurs through cleavage by a disintegrin-and-metalloproteinase-17 (ADAM-17), an enzyme activated by inflammation. It reduces the cellular uptake of TG-loaded lipoproteins, causing their accumulation in circulation; hence the correlation between plasma sLDLR and TG. Soluble LDLR could become a new surrogate marker of inflammation.

## Introduction

Soluble low-density lipoprotein receptor (sLDLR) is the circulating ectodomain of transmembrane LDLR, initially discovered as an interferon-induced protein that exhibited neutralizing activity against vesicular stomatitis virus [1, 2]. Strangely, using sera of healthy adult subjects, others and we have observed a remarkable positive correlation between the level of sLDLR and that of triglycerides (TG) [3, 4]. In a cohort of children suffering of familial hypercholesterolemia and their controls, sLDLR showed correlation with TG as well as with the number of very low-density lipoprotein (VLDL) particles of all sizes, and of large LDL particles [5]. This correlation has largely remained unexplained.

TG circulate in blood primarily as lipoproteins. TG-rich lipoproteins (TRL) include chylomicrons, VLDL, and intermediate density lipoproteins (IDL). TG can be transferred from VLDL to LDL in exchange for cholesteryl ester under the action of cholesterol-ester transfer protein (CETP), forming large LDL which, after TG hydrolysis by hepatic lipase (HL) or lipoprotein lipase (LPL), is converted to medium and small dense LDL [6]. Cells, mostly hepatocytes, clear up TRLs via LDLR family of receptors [(i.e. LDLR, VLDLR, LDL-related proteins (LRP)], as well as via heparan sulfate proteoglycan receptors called syndecans, mostly syndecan-1 (SDC1). Genetic inactivation of SDC1 in mice leads to reduced hepatic clearance and increased plasma levels of TRL [7]. Shedding of the ectodomains of these receptors appears to be a physiological means of limiting the entry of circulating lipoproteins into cells or increasing their abundance in circulation. Deng et al. [8] have shown that inducing inflammation in mice with the bacterial endotoxin lipopolysaccharide (LPS) increased the plasma levels of both shed SDC1 and TG. We presume that shedding of other membrane lipoprotein receptors could occur under such treatment and that their plasma soluble forms would correlate with TG. The concomitance suggests the existence a common shedding mechanism.

### ADAM-17: a common sheddase in inflammation

Ectodomains of many transmembrane receptors normally circulate in body fluids and their levels change under certain pathological conditions [9]. Receptor shedding occurs through cleavage by sheddases of the “A Disintegrin And Metalloproteinase” (ADAM) family, primarily by ADAM-17. Otherwise known as Tumor Necrosis Factor α (TNFα)-Converting Enzyme, ADAM-17 is a zinc-dependent endoproteinase expressed in all organs and tissues [10]. Its substrates include growth factors, adhesion molecules, cytokines and their receptors. It influences many physiological processes including cell proliferation, intercellular communication, tissue regeneration, and inflammation [11]. TNFα is its prototypical ADAM-17 inflammatory substrate. Consistently, mice lacking ADAM-17 in their immune cells survived to LPS-induced fatal inflammatory endotoxemia [12]. One can surmise that any inflammatory condition would lead to increased ADAM-17 activity and the shedding of the ectodomains of many transmembrane receptors, including LDLR. Although this receptor is widely expressed, its main site of expression is the liver. Any significant increase of its circulating soluble form in inflammation will probably emanate from this organ. Cytokines secreted by resident or circulating immune cells are known to stimulate hepatic production of LDLR [13], which, as our hypothesis goes, would be partially shed by activated ADAM-17.

### Hypertriglyceridemia in inflammation

Tissue inflammation leads to greater in situ production and secretion of pro- and anti-inflammatory proteins by infiltrating monocytes or macrophages. Acute inflammation is a hallmark of all infections. The inflammatory response is an innate immune mechanism of fending off infection through, among other mechanisms, production of microbicidal by-products of oxidative stress such as reactive oxygen species and free radicals. However, this response may become pathogenic if it does not subside, i.e. if it becomes chronic. Chronic inflammation is characteristic of metabolic disorders such as atherosclerosis, obesity, diabetes, and non-alcoholic fatty liver disease (NAFLD); it is also a feature of autoimmune diseases such as rheumatoid arthritis, systemic erythematosus, and psoriasis [14].

Hypertriglyceridemia always accompanies infection, irrespective of the infectious agent [15, 16]. A cause for this increase is stimulated hepatic production and secretion of VLDL coupled with diminished clearance of TRLs following a reduction of LPL [17]. The loss of cognate transmembrane receptors through shedding undoubtedly contributes to it. In fact, alterations in plasma lipoproteins following infection are parts and parcels of the innate immune system [18]. Indeed, since lipoproteins and soluble receptors can capture and neutralize microbes, toxins and cytokines, they count among the many anti-inflammatory molecules that help regulate acute inflammation and eventually resolve it.

Metabolic disorders too often result in hypertriglyceridemia [19]. This could stem from reduced cellular uptake of TRL caused by shedding of the ectodomains of cognate receptors by inflammation-activated ADAM-17. For examples, in obesity and NAFLD, the number of infiltrating macrophages increase in white adipose tissue (WAT) and liver, respectively [20, 21], confirming their inflamed state. Moreover, there is a positive correlation between circulating levels of WAT-secreted leptin and inflammatory markers, particularly C-reactive protein (CRP), TNFα, and interleukin (IL)-6 [22]. A recent profiling of a cohort of nearly 118,000 subjects of the Copenhagen City Heart Study and the Copenhagen General Population Study in Denmark has demonstrated a correlation between chronic mild to moderate nonfasting hypertriglyceridemia and markers of inflammation, specifically CRP as well as the blood counts of lymphocytes, neutrophils, and monocytes [23].

### Hypothesis: inflammation may explain the TG-sLDLR correlation

Invoking inflammation as a common inducer of both sLDLR shedding and TG accumulation can explain the remarkable correlation between these molecules in plasma. Inflammation activates ADAM-17, which broadly cleaves off the cell surface receptors of TG-loaded lipoproteins, reducing the clearance of TG. The connecting role of ADAM-17 could be verified by measuring the levels of plasma sLDLR and TG in mice globally deficient for this enzyme, such as the ADAM17^ex/ex^ mice [24], under inflammatory treatment: the increase of both molecules should be attenuated in mutant mice relative to their wild-type counterparts. We suggest that sLDLR be counted among the many markers of inflammation like TNFα, CRP, IL-1β, and IL-6. How it compares to these other markers and in which diseases remains to be determined. Interestingly, plasma sLDLR in our previous study [4], like nonfasting triglyceridemia in the Copenhagen study [23], positively correlates with anthropometric risk factors of chronic inflammation such as gender, age, and body mass index. If shown to be specific and sensitive, sLDLR could serve as a surrogate marker of inflammation in metabolic, immune, and infectious diseases. Since sLDLR is positioned late in the cascade of molecular events initiated by inflammation, it could also be a useful prognostic indicator in anti-cytokine immunotherapies of inflammatory disorders [25].

## Data Availability

Not applicable.

## Abbreviations

ADAM-17: A disintegrin-and-metalloproteinase 17
CETP: Cholesteryl ester transfer protein
CRP: C-reactive protein
HL: Hepatic lipase
IDL: Intermediate density lipoproteins
IL-6: Interleukin 6
LPL: Lipoprotein lipase
LPS: Lipopolysaccharide
LRP: LDL-related protein
NAFLD: Non-alcoholic fatty liver disease
SDC1: Syndecan-1
sLDLR: Soluble low-density lipoprotein receptor
TG: Triglycerides
TNFα: Tumor necrosis factor α
TRL: TG-rich lipoproteins
VLDL: Very low-density lipoproteins
WAT: White adipose tissue
